# Multi-Scale Residual Attention Network for Chinese Speech Recognition Through Collaborative Design

**DOI:** 10.3390/e28091037

**Published:** 2026-09-20

**Authors:** Yuying Li, Hongjie Wan, Akash Sutradhar, Jianing Qin

**Affiliations:** Department of Information Engineering, Beijing University of Chemical Technology, No.15 North Third Ring East Road, Beijing 100029, China; leilvling@gmail.com (Y.L.); splashakash99@gmail.com (A.S.); 2024210660@buct.edu.cn (J.Q.)

**Keywords:** Chinese speech recognition, multi-scale feature modeling, residual convolutional network, attention mechanism, deep learning, ASR

## Abstract

In recent years, the application of multi-scale residuals and attention mechanisms in automatic speech recognition has made some progress. However, existing speech recognition systems still have critical limitations. Many existing systems fail to adequately capture multi-scale speech features, struggling with representation gaps and often relying on inefficient serial attention mechanisms. Additionally, lightweight models, while efficient, often isolate information within grouped architectures, limiting the global feature interaction necessary for speech recognition. To address these persistent challenges, we introduce MSRDA-GSCA, an architecture that synergistically integrates a Multi-Scale Residual Deep Convolutional Attention (MSRDA) network with a Group Shuffled Co-Attention (GSCA) mechanism. MSRDA uses parallel convolutions with varied kernels and dilations, plus adaptive residual connections, to capture diverse temporal dependencies and align heterogeneous features. Its dual-path attention recalibrates channel and spatial information with element-wise max fusion. GSCA employs sequential position-wise channel and spatial attention with intermediate channel shuffling, enabling cross-group interaction and enhancing global feature synergy. Experiments on THCHS-30 and ST-CMDS datasets show that the proposed model outperforms existing methods in Character Error Rate (CER) and Word Error Rate (WER). Ablation studies confirm the complementarity of MSRDA and GSCA. With only 4.65 M parameters, MSRDA-GSCA achieves high recognition accuracy while maintaining low computational complexity for efficient deployment.

## 1. Introduction

Deep learning has significantly advanced Chinese automatic speech recognition (ASR), replacing traditional acoustic models with Deep Neural Networks (DNNs) [1], Convolutional Neural Networks (CNNs) [2], and Recurrent Neural Networks (RNNs) [3], enhancing the modeling of complex acoustic patterns [4]. Recently, end-to-end methods like Connectionist Temporal Classification (CTC) [5], attention mechanisms [6], and the Transformer architecture [7] have simplified system design while improving performance in large-vocabulary tasks. Additionally, self-supervised learning and pre-trained models [8,9] have reduced dependence on large annotated datasets, showing strong generalization in low-resource scenarios [10].

Despite significant progress, challenges persist. Many models rely on single-scale representations, limiting their ability to capture the rich temporal and multi-level acoustic structures in Chinese speech, especially under fast speech, pronunciation variability, and noisy environments [11]. Multi-scale modeling addresses this by capturing features across varying temporal and frequency resolutions [12], but often creates representation gaps not well-managed by conventional residual connections. Additionally, deeper networks face optimization challenges such as gradient vanishing and feature degradation, which residual learning helps alleviate [13]. While attention mechanisms improve performance by focusing on relevant regions [14], serial channel-spatial processing increases computational costs and can suppress important features. Grouped architectures, though efficient, tend to isolate information across channels, limiting global feature interaction.

To address these limitations, we propose MSRDA-GSCA, a model integrating multi-scale residual deep convolutional attention (MSRDA) with a group shuffled co-attention (GSCA) mechanism. The MSRDA module uses parallel multi-scale dilated convolutions to capture both local and long-range dependencies, with an adaptive residual connection that bridges heterogeneous feature representations and stabilizes training. A dual-path attention mechanism simultaneously models channel and spatial information, using a max-response fusion strategy. The GSCA module introduces a co-attention pipeline with channel shuffle, enabling cross-channel information exchange and reducing feature isolation.

Before detailing our specific contributions, it is essential to position the methodological novelty of this work. While foundational mathematical operators such as 1×1 projections, channel shuffling, and spatial attention are individually established in general deep learning, the core innovation of this paper lies in their problem-specific collaborative design tailored for Mandarin acoustic modeling. Existing speech recognition networks often treat multi-scale feature extraction and attention-based recalibration as isolated, sequential steps, which can lead to representation bottlenecks. In contrast, our proposed architecture structurally couples these mechanisms to achieve synergistic representation learning. Specifically, the MSRDA module is designed to capture diverse time–frequency granularities while utilizing a max-based fusion mechanism to actively preserve dominant acoustic energy peaks (e.g., critical formant transitions) that are typically diluted by standard averaging or concatenation. Concurrently, the GSCA module is explicitly engineered to overcome the “channel-isolation” bottleneck inherent in highly efficient lightweight grouped convolutions. By seamlessly integrating these components, the MSRDA-GSCA framework provides a collaborative architectural solution that balances computational efficiency with comprehensive multi-granularity acoustic representations.

The main contributions of this paper are summarized as follows:

1. Multi-Scale Residual Deep Convolutional Attention (MSRDA) Module: We propose a novel MSRDA module, which uses parallel 1 × 1, 3 × 3, 5 × 5, and dilated 3 × 3 convolutions. Combined with an adaptive projective residual connection, this architecture bridges representation gaps and broadens the receptive field without increasing model complexity.
2. Parallel dual-path attention with max-response fusion: Unlike traditional serial attention mechanisms, we introduce a lightweight parallel attention design. By concurrently recalibrating channel and spatial information with a max-response fusion strategy, the model preserves key acoustic features while maintaining a minimal parameter footprint.
3. Group shuffled co-attention (GSCA) module: To mitigate feature isolation in grouped architectures, we design the GSCA module. It integrates a dual-attention pipeline with channel shuffle to enable cross-channel information exchange, enhancing global feature interaction and discriminative power.
4. Experimental Validation: We conduct comparative experiments and ablation studies on the THCHS-30 and ST-CMDS datasets to validate the model’s performance and module contributions.

The structure of the following sections is outlined below:

Section 2 covers the foundational theories of MSCNNs, residual learning, and attention mechanisms in modern speech recognition. Section 3 introduces the MSRDA-GSCA module, our core contribution. Section 4 presents extensive experiments and analysis to evaluate the proposed model. Finally, Section 5 summarizes the entire work and offers concluding remarks.

## 2. Related Work

### 2.1. Multi-Scale Convolutional Neural Networks (MSCNNs)

Multi-scale CNNs [15] improve speech recognition by using architectures with diverse receptive field sizes to capture temporal and frequency variability. CNNs outperformed traditional DNNs in LVCSR by reducing sensitivity to frequency variations, with a 6–10% error reduction on TIMIT [16]. Later models like Big-Little Net [17] and Multi-Scale Octave Convolutions [18] introduced parallel branches and decomposed feature maps to balance accuracy and efficiency. End-to-end models such as Conformer and SA-MSTCN [7,19], integrate multi-scale convolutions with attention mechanisms, improving WER. However, fixed-kernel models still struggle with adapting to speech variability, limiting their ability to balance phonetic detail and global context.

### 2.2. Residual Learning Architectures

Since the introduction of Residual Networks (ResNet) [20], residual learning has become a key method in deep acoustic models. It addresses challenges like gradient vanishing and performance degradation by using identity-mapped skip connections, enabling deep network training. Residual connections facilitate gradient flow and improve optimization, supporting deeper architectures [21,22]. Architectural refinements, like bottleneck blocks, further enhance efficiency and scalability [20,23]. The Residual Attention Gated Linear Unit (RAGLU) [24] combines channel and temporal attention to improve speech clarity. However, residual networks still face limitations, such as redundancy, lack of scale awareness, and difficulty aligning multi-scale features, limiting their ability to efficiently fuse hierarchical representations.

### 2.3. Attention Mechanisms in Speech Recognition

Attention mechanisms are vital in ASR for modeling long-range dependencies and enhancing feature representation. Self-attention and multi-head attention, used in encoder–decoder architectures, improve the mapping between speech inputs and text outputs, reducing errors compared to traditional alignment methods [25]. Transformer-based models, such as Conformer, leverage self-attention to capture global context, often outperforming RNNs and CNNs on long sequences [7,26]. Variants like channel and spatial attention have been developed for time–frequency learning, where channel attention emphasizes informative frequency components [27] and spatial attention improves noise robustness [28]. Cross-channel attention boosts information fusion in multi-speaker or multi-channel scenarios [29], while hybrid attention mechanisms improve generalization in noisy or low-resource environments [30]. However, existing ASR attention models fail to address inter-channel dependencies, leading to redundancy, suppressed cues, and information isolation in grouped models, limiting efficient and hardware-friendly feature recalibration.

## 3. MSRDA-GSCA Model

**Figure 1:**
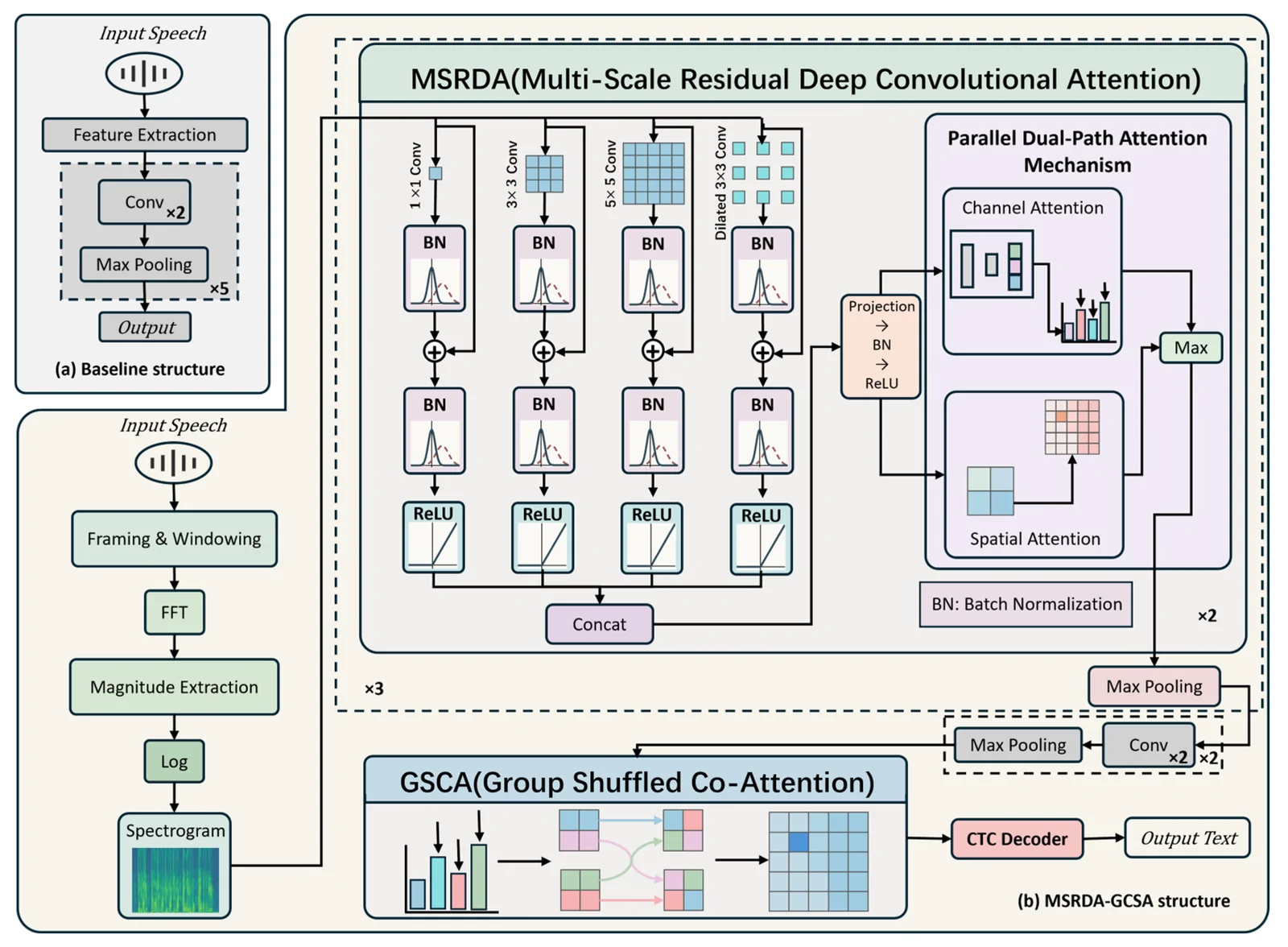
Overall framework of the speech recognition system based on MSRDA-GSCA, including (**a**) the baseline structure and (**b**) the proposed multi-scale residual deep convolutional attention with group shuffled co-attention (MSRDA-GSCA) structure.

Traditional speech recognition systems, as exemplified by the DCNN-CTC [31] architecture in Figure 1a, primarily execute the mapping from acoustic features to pinyin sequences through feature encoding and CTC-based decoding. However, they struggle to capture long-term context. Fixed convolution kernels cannot handle complex speech fluctuations, while uniform feature processing fails to focus on key information. These limitations hinder overall recognition accuracy. We propose a model integrating multi-scale residual deep convolutional attention networks with a group shuffled co-attention mechanism (MSRDA-GSCA). Figure 1b illustrates the MSRDA-GSCA framework, an architecture designed to integrate multi-scale residual convolutions with global attention mechanisms. The comprehensive structure of this model is characterized by the synergistic operation of two core components: the MSRDA fusion module and the GSCA module. Specifically, the MSRDA module is engineered to facilitate the adaptive extraction of multi-granularity time–frequency features while simultaneously mitigating optimization challenges, such as gradient vanishing, via adaptive projection residual learning paths. Building upon these enriched representations, the GSCA module performs a precise recalibration of feature importance across both channel and spatial dimensions. By suppressing redundant acoustic variability and amplifying critical discriminative cues, this dual-attention mechanism ensures high fidelity in complex acoustic environments. The proposed system effectively bridges the gap between local feature extraction and global context modeling, thereby enhancing overall recognition performance.

### 3.1. MSRDA Module

**Figure 2:**
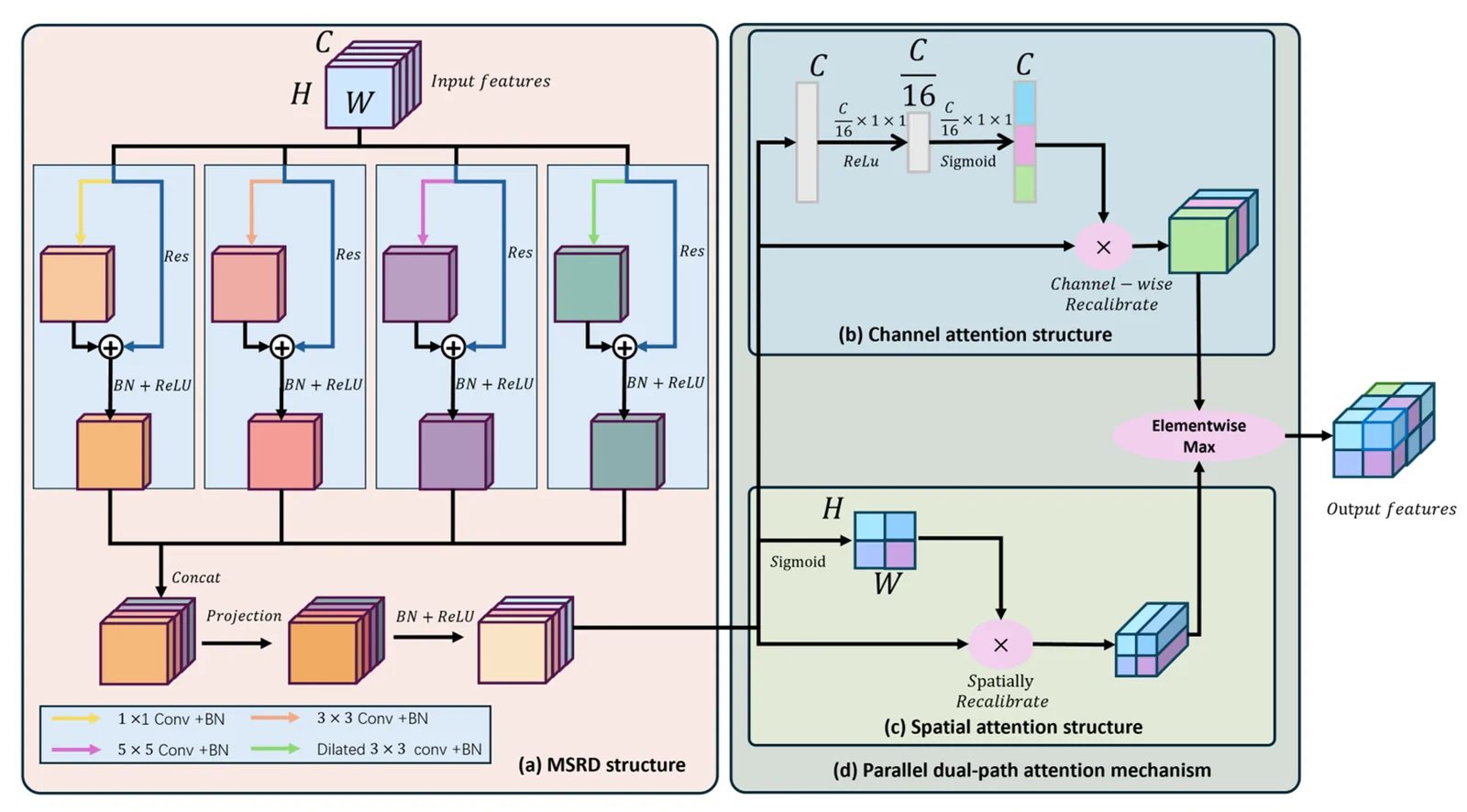
Detailed architecture of the proposed MSRDA module, consisting of (**a**) the Multi-Scale Residual Deep Convolution (MSRD) structure for diverse feature extraction, (**b**) the channel attention branch, (**c**) the spatial attention branch, and (**d**) the parallel dual-path attention mechanism for feature recalibration.

The internal structure of the proposed multi-scale residual deep convolutional attention networks (MSRDA) module is shown in Figure 2. This module combines multi-scale feature extraction with residual learning to improve the representation accuracy of complex acoustic features. Specifically, the MSRDA module uses parallel convolutional branches with different receptive fields. This allows it to capture time–frequency characteristics at multiple granularities. The parallel setup overcomes the limitations of fixed-scale kernels, enabling the model to process short-term details and long-term context information simultaneously. To ensure stable optimization in deep architectures, an adaptive projection residual block is proposed. These connections use identity mapping paths to ease the problem of gradient vanishing, thus smoothly transmitting low-level acoustic cues to deeper layers. Moreover, the deep attention mechanism adaptively adjusts the fused multi-scale features. By assessing correlations in both channel and spatial dimensions, it makes the model focus on highly discriminative speech areas and reduce environmental interference. As a result, the MSRDA module greatly enhances the model’s ability to capture features through the combined effect of multi-scale perception and attentional control.

#### 3.1.1. Multi-Scale Deep Convolution (MSD)

Traditional DCNN architectures often use single-scale convolution kernels, which restrict feature extraction. Speech signals, however, change over time and have features across various temporal and frequency scales. For example, individual phonemes and syllables show up in short-term, high-frequency information. Meanwhile, prosody, emotion, and context need longer-term modeling. Multi-scale fusion convolution uses kernels of different sizes. This lets the network extract features at multiple scales, capturing both short- and long-term speech traits. It boosts the model’s ability to represent features, helping it catch fine details and context and thus improving recognition accuracy.

Moreover, by integrating features across different scales, multi-scale convolution mitigates the impact of irrelevant acoustic variations. This comprehensive integration improves the model’s adaptability to diverse acoustic environments and yields highly resilient representations. As illustrated in Figure 2a, the proposed deep multi-scale fusion convolution architecture is composed of 4 groups of convolutional blocks. Each group contains 4 parallel convolution branches at different scales. The 1 × 1 and 3 × 3 small-kernel convolutions are used to capture fine-grained speech features, while the 5 × 5 large-kernel convolution is designed to capture information over longer temporal scales. Additionally, a 3 × 3 dilated convolution (with a dilation rate of 2) is introduced to further expand the receptive field and capture broader contextual dependencies. These features are fused and projected back to the target channel dimension via a 1 × 1 convolution and then sent to subsequent layers for further processing.

For an input speech feature map $X\in R^{H\times W\times C}$, where H denotes the temporal dimension (i.e., the number of frames), W represents the frequency dimension (e.g., Mel-frequency bins or STFT frequency bins), and C is the number of input feature channels. The convolution operation of each branch is defined as

(1) $$Y_{i}=ReLU(BN\left(Conv\left(X,K\right)\right))$$

where Conv(⋅) denotes the convolution operation, *K* represents the convolution kernel, and $Y_{i}$ is the resulting feature map. By employing convolution kernels of different sizes (e.g., 1 × 1, 3 × 3, and 5 × 5), each branch extracts information at a distinct scale.

Dilated convolution enlarges the receptive field by inserting gaps between the elements of the convolution kernel. In speech recognition, dilated convolution enables the modeling of long-range temporal dependencies. The dilated convolution operation can be formulated as

(2) $$Y_{dilated}\left(t\right)=\sum_{i=0}^{k-1}X\left(t+i\cdot d\right)\cdot K_{i}$$

where X(t) denotes the input feature map, $K_{i}$ represents the i-th element of the convolution kernel, d is the dilation rate, k denotes the kernel size, and $Y_{\mathrm{dilated}}(t)$ is the output of the dilated convolution. The convolution kernel/dilation rate configuration used here is (k, d) = (3, 2).

Each branch applies Batch Normalization (BN) followed by ReLU activation after the convolution operation. BN accelerates training and improves stability by normalizing the inputs of each layer to have zero mean and unit variance. The BN operation is defined as

(3) $$\hat{X}=\frac{X-\mu }{\sqrt{\sigma^{2}+\epsilon }}$$

where μ and $\sigma^{2}$ denote the mean and variance of the input, respectively, and ϵ is a small constant introduced to prevent division by zero. After normalization, the output is scaled and shifted using learnable parameters γ and β.

The ReLU activation function is defined as

(4) $$ReLU\left(X\right)=\max\left(0,X\right)$$

Finally, the outputs of the four branches are concatenated through a Concatenate layer to form a fused feature map. Let the outputs of the four branches be denoted as $Y_{1}$, $Y_{2}$, $Y_{3}$, and $Y_{dilated}$, respectively. The concatenated feature map is given by

(5) $$Y_{\mathrm{cat}}=Concat\left(Y_{1},Y_{2},Y_{3},Y_{dilated}\right)$$

While the mathematical formulation of the adaptive projective residual shares structural similarities with conventional projection shortcuts (such as those employed in standard ResNets), its technical novelty fundamentally lies in its distinct architectural placement and functional application within our framework. In traditional multi-branch networks, a residual connection typically wraps around the entire composite block. However, acoustic features extracted from highly heterogeneous receptive fields (i.e., 1×1, 3×3, 5×5, and dilated convolutions) capture vastly different time–frequency dynamics. Forcing these disparate representations to fuse solely at the terminal end of the multi-scale block often induces severe gradient conflicts and early-stage feature suppression. To address this, our design embeds the adaptive projection residual internally within each independent parallel branch. This localized placement forces each specific scale to learn a residual correction directly relative to the original acoustic input. Consequently, this branch-level integration prevents the suppression of fine-grained acoustic cues and stabilizes the joint optimization of multi-resolution acoustic dynamics during the early phases of training.

#### 3.1.2. Adaptive Projection Residual Block

**Figure 3:**
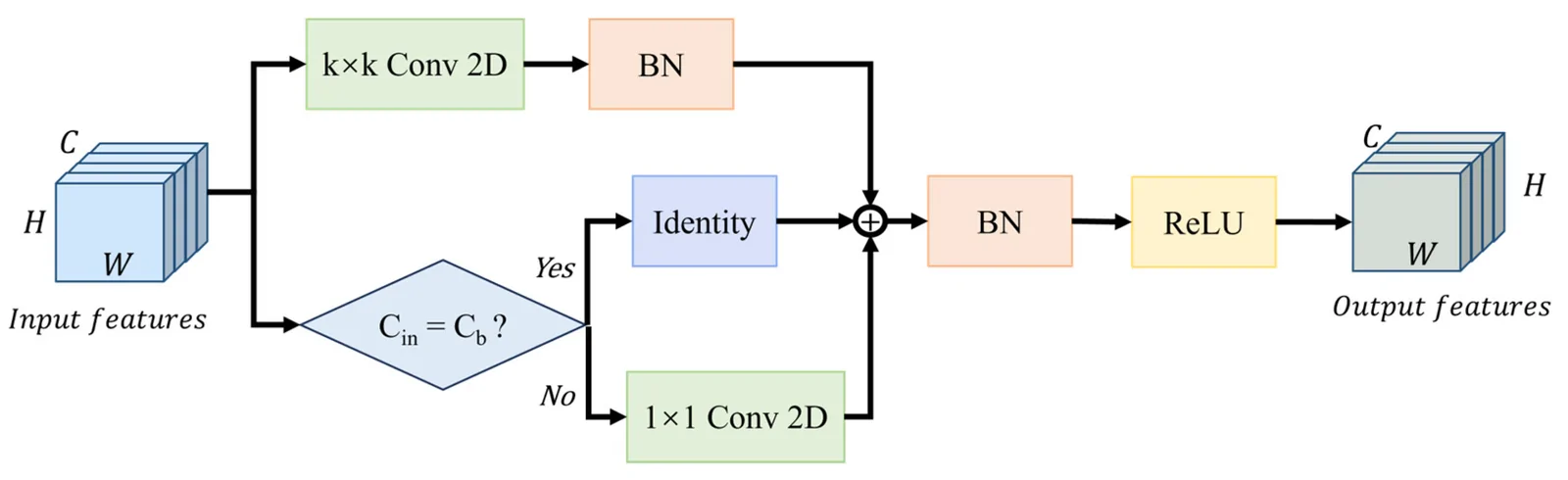
Adaptive projection residual block structure.

In deep neural networks, increasing network depth often leads to issues such as gradient vanishing, feature degradation, and training instability. Figure 3 illustrates that adaptive projection residual learning introduces an identity mapping, enabling the network to learn residual information relative to the input features rather than directly learning the target mapping H(X). Specifically, the residual function is defined as F(X)=H(X)−X.

Accordingly, the final output can be expressed as

(6) $$Y=X+F\left(X\right)$$

This structure explicitly provides a shortcut path for information and gradient flow, thereby effectively alleviating optimization difficulties in deep models.

In the proposed multi-scale convolution module, each convolutional branch incorporates a residual connection, and its output can be uniformly formulated as

(7) $$B_{i}=ReLU({BN}_{2}({BN}_{1}\left({Conv}_{i}\left(X\right)\right)+{Res}_{i}\left(X\right)))$$

where $X\in R^{H\times W\times C}$ denotes the input acoustic feature tensor, ${Conv}_{i}(\cdot )$ represents the convolutional mapping at the i-th scale, BN(⋅) denotes the batch normalization operation, and ${Res}_{i}(\cdot )$ is the residual mapping function.

When the input and the branch output have the same number of channels, ${Res}_{i}(\cdot )$ degenerates into an identity mapping. When the channel dimensions are inconsistent, a 1×1 convolution is employed to linearly project the input features for channel alignment.

Unlike approaches that introduce residual connections only outside the entire multi-scale module, this work incorporates residual connections within each convolutional branch. This design is of particular importance for the following reasons: (1) each scale branch directly references the input features; (2) each branch learns a scale-specific residual correction relative to the original features; and (3) it prevents any single scale branch from exerting excessive interference on the overall feature representation during early training. This branch-level residual design enables more independent and stable multi-scale feature learning.

In multi-scale architectures, the output channel dimensions of different branches are typically inconsistent. If identity mappings are directly applied for residual addition, dimension mismatch issues may arise. To address this problem, a 1×1 convolutional projection is introduced along the residual path:

(8) $${Res}_{i}\left(X\right)=\left\{\begin{array}{cc} X, & C=C_{i}\\ {Conv}_{1\times 1}\left(X\right), & C\neq C_{i} \end{array}\right.$$

This design not only resolves the channel alignment issue but also allows the model to perform lightweight linear transformations along the residual path, thereby enhancing representational flexibility.

Residual connections shorten the gradient path, enabling error signals to bypass nonlinear transformations and directly update shallow layer parameters. This is critical in multi-branch, multi-scale architectures, where parallel convolutional (e.g., 1×1, 3×3, 5×5, and dilated 3×3) can cause gradient instability and feature misalignment. Standard identity mapping fails to bridge gaps between heterogeneous feature streams with different distributions and receptive fields. We introduce an Adaptive Projective Residual mechanism, using a learnable projection to align input and multi-scale outputs. In speech recognition, this preserves low-level acoustic cues while enabling effective fusion with high-level semantic representations, stabilizing training and improving multi-scale feature synergy.

#### 3.1.3. Parallel Dual-Path Attention Mechanism

In order to simultaneously capture the importance of feature maps in the channel dimension and the key regions in the spatial dimension, this paper designs a parallel dual-path attention mechanism, as shown in Figure 2d. This module consists of three parts: channel attention, spatial attention, and feature fusion.

The architecture of the channel attention module is illustrated in Figure 2b. It aims to model the dependencies between feature channels. Given the input feature map $U\in R^{H\times W\times C}$, it is first compressed into a vector $z\in R^{1\times 1\times C}$ by Global Average Pooling to obtain the global receptive field. Subsequently, a nonlinear transformation is performed through two layers of 1×1 convolutional layers:

(9) $$U_{t}=U⊗Sigmoid(f_{c2}(ReLU(f_{c1}(GAP\left(U\right)))))$$

where ⊗ denotes element-wise multiplication across channels, $GAP\left(\cdot \right)$ represents the Global Average Pooling operation, $f_{c1}$ and $f_{c2}$ denote the first and second 1×1 convolutional layers (acting as fully connected layers), where $f_{c1}$ reduces the channels to C/r and $f_{c2}$ restores them to C.

The Sigmoid activation function is defined as

(10) $$Sigmoid\left(X\right)=\frac{1}{1+e^{-x}}$$

Unlike channel attention, spatial attention focuses on identifying regions in the image that contribute more meaningfully. This branch directly applies a convolutional layer with a kernel size of 1×1 to the input feature U, projecting the multi-channel information into a single-channel spatial map, which is then normalized by a Sigmoid function to obtain the spatial mask $M_{s}\in R^{H\times W\times 1}$. This process enhances the geometric structure information of the target:

(11) $$U_{k}=U⊗Sigmoid\left(f_{conv1\times 1}\left(U\right)\right)$$

where $f_{conv1\times 1}$ denotes a 1×1 convolution layer with a single filter and $U_{k}$ is the output where spatial position is re-weighted across all channels.

In this study, a parallel competition mechanism was used to merge the above two branches. Unlike traditional serial joins (such as CBAM), this paper uses the element-wise maximum operation to aggregate the channel reinforcement feature $U_{t}$ and the space enhancement feature $U_{k}$:

(12) $$U_{out}=\max\left(U_{t},U_{k}\right)$$

where $\max\left(\cdot \right)$ denotes the element-wise maximum operator, which preserves the most salient activations from either branch. The advantage of using maximum lies in its ability to preserve the most prominent activation features in two dimensions in a non-linear manner, avoiding signal attenuation that may result from consecutive multiplication, thereby enabling the model to have stronger feature recognition capability in complex backgrounds.

#### 3.1.4. Summary of the MSRDA Module

The proposed module is not a simple stacking of multi-scale convolutions, residual connections, and attention mechanisms; rather, it is a collaboratively designed architecture tailored to the acoustic characteristics of Chinese speech recognition. Each component addresses modeling challenges at a different level and forms a complementary relationship within the overall framework. Multi-scale convolutions capture diverse acoustic patterns but bring problems like conflicting responses and gradient instability. Residual connections, crucial for this module, act as a stable path. They not only improve training controllability by making the model learn complementary information but also enhance the capture of acoustic dynamics, in line with traditional speech dynamic-feature use, vital for Chinese tonal recognition. The parallel dual-path attention mechanism, through coordinated channel and spatial attention and a max-based strategy, boosts acoustic feature discriminability, efficiently capturing key information. Overall, these components work together to optimize Chinese speech recognition.

### 3.2. Group Shuffled Co-Attention Module (GSCA)

In traditional DCNNs, convolution operations mainly focus on modeling local receptive fields, while paying limited attention to global inter-channel dependencies and the importance differences across spatial dimensions. However, in ASR tasks, acoustic features contain not only local time–frequency structures but also global correlations spanning multiple time steps and frequency bands. For example, tonal contours, syllabic structures, and spectral energy distributions often require joint modeling over a broader temporal and spectral context. Neglecting such global dependencies in the channel and spatial dimensions constrains the model’s ability to capture high-level acoustic semantics, thereby degrading recognition performance.

**Figure 4:**
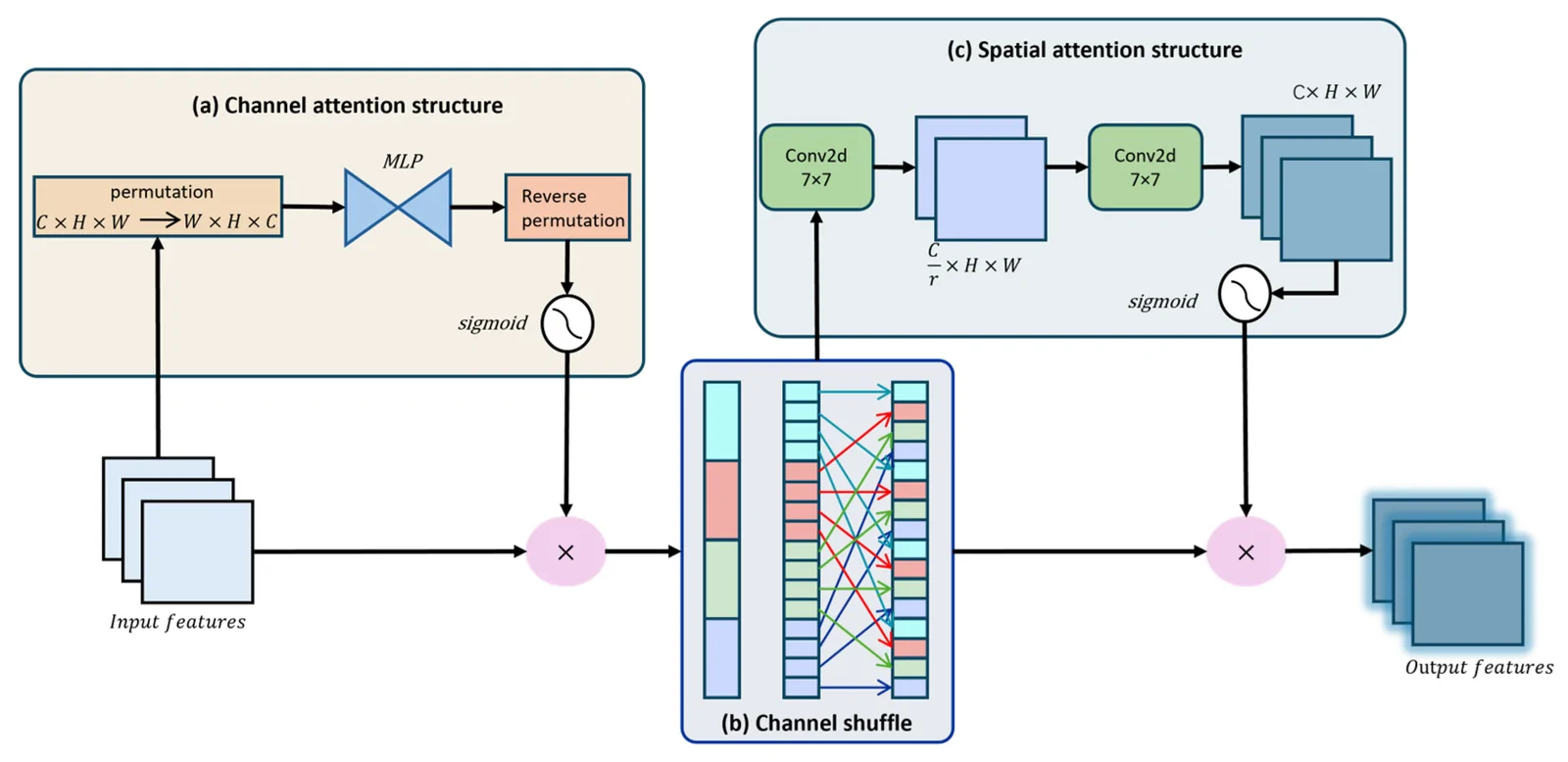
Detailed structure of the GSCA module, comprising (**a**) the permutation-based channel attention, (**b**) the channel shuffle operation for information interaction, and (**c**) the spatial attention branch for global context aggregation.

To address this limitation, this paper proposes a Group Shuffled Co-Attention Module (GSCA) module, as illustrated in Figure 4. Through the coordinated design of channel attention modeling, channel shuffle, and spatial attention modeling, the proposed module effectively enhances the global representational capacity of feature maps while maintaining computational efficiency.

#### 3.2.1. Position-Wise Channel Attention Modeling (PCA)

In convolutional acoustic models, different channels often correspond to distinct acoustic subspaces, such as features that are more sensitive to energy variations in specific frequency bands, temporal context, or spectral shapes. Therefore, explicitly modeling inter-channel dependencies helps the network adaptively emphasize more discriminative acoustic subspaces.

As illustrated in Figure 4a, let the input feature map be $X\in R^{H\times W\times C}$, where C denotes the number of channels, and H and W represent the temporal and frequency dimensions, respectively. The input feature map is first permuted to move the channel dimension to the last axis, facilitating inter-channel relationship modeling. Subsequently, a bottleneck structure composed of a two-layer multilayer perceptron (MLP) is introduced to learn channel dependencies: the first layer compresses the channel dimension to one-fourth of the original size and applies a ReLU activation to introduce nonlinearity; the second layer restores the channel dimension to its original size. Finally, a Sigmoid activation function generates the channel attention weights. The enhanced feature map is obtained by position-wise multiplication of the input feature map with the channel attention map:

(13) $$F_{channel}=Sigmoid(MLP(Permute(F_{input})))⊗F_{input}$$

where $F_{\mathrm{channel}}$ denotes the enhanced feature map, ⊗ represents position-wise multiplication, and $F_{\mathrm{input}}$ is the original input feature map. This design allows the model to selectively amplify discriminative channels while suppressing less informative ones.

#### 3.2.2. Channel Shuffling and Global Information Mixing (CSM)

Relying solely on channel attention may be insufficient to fully facilitate inter-channel information interaction. To further enhance the information fusion capability among different acoustic subspaces, a channel shuffle operation is introduced after the channel attention, as illustrated in Figure 4b. Specifically, the enhanced feature map is first divided along the channel dimension into g groups (in this work, g=4), with each group containing C/g channels. The grouped features are then transposed and permuted and the original tensor shape is restored while preserving the permuted channel order. This operation breaks the fixed grouping of channels, enabling features across different groups to interact effectively in subsequent convolutional layers, thereby improving the diversity and global consistency of the feature representation.

The channel shuffle process can be formally expressed as

(14) $$F_{shuffle}=ChannelShuffle\left(F_{channel}\right)$$

where $F_{\mathrm{shuffle}}$ denotes the shuffled feature map and $F_{\mathrm{channel}}$ is the input feature map after channel attention.

#### 3.2.3. Spatial Attention Modeling (SAM)

In addition to the channel dimension, different locations on the time–frequency plane of speech features also exhibit pronounced variations in importance. For example, vowel steady-state regions, consonant burst regions, and tonal variation intervals are usually concentrated in specific time–frequency areas, whereas redundant acoustic features often appear in localized or band-shaped distributions. Therefore, it is necessary to explicitly model the spatial dimension.

As shown in Figure 4c, in the spatial attention submodule, the input feature map is first processed by a 7×7 convolutional layer, which reduces the number of channels to one quarter of the original. This is followed by batch normalization and a ReLU activation to introduce nonlinearity. Subsequently, a second 7×7 convolutional layer is applied to restore the channel dimension to the original size C, followed by another batch normalization layer. Finally, a Sigmoid activation function is used to generate the spatial attention map. The shuffled feature map and the spatial attention map are then multiplied element-wise to obtain the final output feature map:

(15) $$F_{spatial}=Sigmoid(Conv(BN(ReLU(Conv(F_{shuffle})))))⊗F_{shuffle}$$

where $F_{\mathrm{spatial}}$ denotes the feature map after spatial attention.

The final output feature map thus contains enhanced representations obtained through the successive application of channel attention, channel shuffle, and spatial attention.

#### 3.2.4. Summary of the GSCA Module

In summary, the proposed GSCA module achieves global dependency modeling and fine-grained selection of acoustic features through the collaborative design of point-wise channel attention, channel shuffle, and spatial attention. Channel attention focuses on modeling which acoustic subspaces are more important, channel shuffle facilitates cross-channel information interaction, and spatial attention emphasizes where key acoustic events occur in the time–frequency domain. These three components complement each other, enabling the model to enhance the joint modeling of global and local acoustic information while maintaining a relatively low computational overhead, thereby providing an effective feature enhancement mechanism for high-performance speech recognition.

### 3.3. Complete Network Configuration

All output sizes in Table 1 omit the batch dimension. All convolutions have a stride of 1 and the same padding. The three size-reducing pooling layers have 2 × 2 kernels, stride 2, and valid padding. The two later 1 × 1 pooling layers preserve dimensions.

Attention configurations and residual ordering are shown in Table 2. Branch, projection, fusion, and later backbone convolutions use He-normal initialization. The branch and fusion convolutions omit biases; the four later 7 × 7 backbone convolutions include biases. Backbone BN layers explicitly use epsilon 0.0002. GSCA BN layers retain their implementation defaults.

## 4. Experiments

### 4.1. Dataset Selection and Evaluation Metrics

#### 4.1.1. Dataset Selection

To evaluate the performance of the proposed MSRDA-GSCA model, two public Chinese speech corpora, THCHS-30 [32] and ST-CMDS [33], were employed. THCHS-30, released by the Center for Speech and Language Technology of Tsinghua University, contains over 30 h of high-quality read speech and provides supplementary resources such as language models, pronunciation lexicons, and noisy test sets, making it suitable for baseline training. ST-CMDS, released by SurfingTech, contains approximately 100 h of mobile-recorded speech from 855 speakers and mainly consists of daily commands and search queries. Compared with the read speech in THCHS-30, ST-CMDS is more suitable for evaluating model generalization in task-oriented and mobile speech scenarios. The technical specifications of the two corpora are summarized in Table 3. Their combination enables a more comprehensive evaluation of the model under both formal and informal acoustic conditions.

THCHS-30 used the official training/development/test partition of 10,000/893/2495 utterances. ST-CMDS used a random utterance partition containing 100,000 training utterances, 600 development utterances, and 2000 test utterances, totaling 102,600. The corpus descriptions and nominal partition sizes are separated in Table 3 so that corpus scale is not confused with the size of an individual split. ST-CMDS contains 855 speakers at the corpus level. Its random utterance partition should not be described as speaker-independent, because speaker-disjointness has not been established. Recordings from the same speaker may therefore occur in different splits. Accordingly, this protocol does not support a claim of generalization to entirely unseen speakers.

#### 4.1.2. Evaluation Metrics

CER is one of the most commonly used metrics for evaluating the performance of text recognition and speech recognition systems, and it measures the degree of discrepancy between the predicted text and the reference text at the character level [34]. CER is based on the edit distance (Levenshtein Distance) and is computed by counting character-level substitutions (Substitution), insertions (Insertion), and deletions (Deletion). The calculation is defined as follows:

(16) $$CER=\frac{S_{c}+D_{c}+I_{c}}{N_{c}}$$

where $S_{c}$ denotes the number of character substitutions, $D_{c}$ denotes the number of character deletions, $I_{c}$ denotes the number of character insertions, and $N_{c}$ denotes the total number of characters in the reference text. A lower CER indicates higher recognition accuracy at the character level. This metric is widely adopted in languages without explicit word boundaries, such as Chinese and Japanese, as well as in optical character recognition (OCR) tasks.

WER is one of the most widely used evaluation metrics in speech recognition and natural language processing, and it measures the recognition error rate at the word level [35]. Similar to CER, WER is also based on edit distance, but the basic unit of operation is the “word” rather than the character. The calculation is defined as follows:

(17) $$WER=\frac{S_{w}+D_{w}+I_{w}}{N_{w}}$$

where $S_{w}$ denotes the number of word substitutions, $D_{w}$ denotes the number of word deletions, $I_{w}$ denotes the number of word insertions, and $N_{w}$ denotes the total number of words in the reference text. WER provides a more intuitive reflection of recognition performance at the level of semantic units (words) and is therefore most commonly used in tasks where words serve as the basic units, such as English speech recognition.

The Real-Time Factor (RTF) is employed as the primary metric for evaluating inference efficiency, defined as the ratio of the total inference time to the total duration of the processed speech signal. A lower RTF value signifies higher computational efficiency and better suitability for real-time deployment:

(18) $$RTF=\frac{T_{inference}}{T_{audio}}$$

where $T_{inference}$ denotes total time taken by the model to complete inference (unit: seconds), this usually refers to the time from inputting acoustic features to outputting recognition results. And $T_{audio}$ denotes the actual physical duration of the processed audio (in seconds).

#### 4.1.3. Acoustic Features and Data Augmentation

The acoustic representation is a log-magnitude spectrogram. At a sampling rate of 16 kHz, frames use a 25-ms Hamming window (400 samples) and a 10-ms hop (160 samples). A 400-point FFT is applied to each windowed frame. The first 200 magnitude coefficients are retained and compressed using ln(1 + magnitude). The extraction routine does not apply additional feature-level mean-variance normalization.

The configured training entry point uses the SpecAugment feature class. The development-set evaluation and single-file recognition entry points use the unaugmented Spectrogram class.

The masking routine selects one of four mutually exclusive branches. Table 4 states their actual behavior. Mask widths are uniformly sampled from the integers 1 through 100; starts are uniformly sampled from 1 through the relevant dimension length. Array slicing clips the upper boundary and can produce shortened or empty masks. Selected feature values are set to zero.

The fourth branch uses the expression [$:v_{start}:v_{start}+v_{width}$] on the frequency axis. Its positive step exceeds its stop, so it selects the first frequency bin rather than performing the intended combined mask. This is reported as an implementation detail, not as a newly designed augmentation. No rerun with a changed masking expression is claimed.

#### 4.1.4. Acoustic Decoding and Pinyin-to-Character Conversion

The decoding pipeline of the proposed system is explicitly decoupled into a purely acoustic CTC decoding stage and an application-level text-conversion stage.

First, the acoustic classifier produces 1428 class probabilities per subsampled time step, corresponding to a vocabulary of 1427 tone-marked pinyin symbols and one CTC blank label. During this acoustic phase, standard CTC greedy decoding is employed to generate the pinyin sequence. To strictly evaluate the raw representational capability of the acoustic architecture, no external language model rescoring is applied to the frame-level acoustic posteriors.

The separate text-conversion stage translates the decoded pinyin symbols into Chinese characters using a dictionary-constrained character-bigram beam search. Each predicted pinyin token generates a set of candidate characters from a pronunciation dictionary. Ambiguous pinyin-to-character mappings are dynamically resolved using transition probabilities. Specifically, extending a sequence from character $c_{t-1}$ to $c_{t}$ updates the path score by multiplying the current sequence score by the conditional probability $\frac{N\left(c_{t-1},c_{t}\right)}{N\left(c_{t-1}\right)}$, where N denotes the statistical bigram or unigram counts. The expanded candidates are ranked and strictly pruned to a beam width of 100. Unseen bigrams are excluded without unigram back-off smoothing. If no valid continuation survives, the decoder commits the preceding optimal segment, resets its state, and attempts to initialize from the current token. A pinyin token absent from the dictionary produces no candidates and is ignored.

Finally, because continuous Chinese text lacks explicit word boundaries, evaluating the Word Error Rate (WER) requires a standardized tokenization step. The widely adopted Jieba segmentation tool is systematically applied to segment both the final predicted Chinese character sequences and the ground-truth transcripts into discrete words before calculating the final WER metrics.

### 4.2. Experiment Setup

All experiments in this study were conducted on a standardized computing platform. The detailed hardware specifications, including the Intel Core i5-12400F CPU and NVIDIA GeForce RTX 3060 GPU, along with the software environment (e.g., TensorFlow 2.8.4), are summarized in Table 5.

To ensure optimal convergence and generalization, a specific set of hyperparameters was employed during the training phase, as detailed in Table 6. Considering the high temporal resolution of Mandarin speech (up to 1600 frames per sample) and the inherent parallelism of the MSRDA-GSCA architecture, we selected a Batch Size of 4. This choice optimizes the trade-off between gradient stability and the massive memory footprint required by the simultaneous multi-scale feature recalibration and parallel dual-path attention mechanisms. The reported results for the in-house experiments are based on one training run per configuration.

### 4.3. Comparative Experiments on THCHS-30

To verify the effectiveness of the proposed method, comparative experiments are conducted against several representative and widely used approaches, including the DCNN+CTC model (baseline) [31], the DFSMN-3-gram model [36], the TCN-Transformer-CTC model [37], the DFCNN-Transformer model [38], the Deep Speech 2 model [34], and the LST-RPESR model [35]. Table 7 summarizes the experimental results of the comparative evaluations. From the experimental results, it can be observed that the baseline DCNN+CTC model achieves a CER of 15.45% and a WER of 20.67% on the THCHS-30 dataset. In contrast, the proposed MSRDA-GSCA model reduces the CER and WER to 5.94% and 8.32%, respectively, achieving substantial performance improvements on both evaluation metrics. Compared with the baseline, the CER is reduced by 9.51 percentage points and the WER by 12.35 percentage points, indicating that the proposed method exhibits clear advantages in both character-level and word-level recognition.

Compared with other selected reference architectures, MSRDA-GSCA also demonstrates good recognition performance. In comparison to the LST-RPESR model, the CER is further reduced by 7.83 percentage points and the WER by 12.34 percentage points. As opposed to Transformer-based approaches, such as TCN-Transformer-CTC and DFCNN-Transformer, the proposed model achieves larger improvements on both metrics. These results suggest that relying solely on temporal modeling or global modeling is insufficient to fully capture the multi-scale characteristics of speech signals. The performance gains can be mainly attributed to the introduction of the multi-scale residual attention deep convolutional structure in MSRDA-GSCA, which effectively extracts both local and contextual speech features under different receptive field scales. In addition, the GSCA modules further enhance the model’s capability to focus on critical information regions while suppressing redundant features, thereby improving overall recognition accuracy.

Based on the comparative experimental results on the THCHS-30 dataset, the following conclusions can be drawn: (a) compared with the baseline DCNN+CTC model, the proposed MSRDA-GSCA achieves certain improvements in both CER and WER, validating the effectiveness of the proposed model design; (b) the multi-scale residual attention deep convolutional structure enables more comprehensive modeling of multi-level speech features and plays an important role in reducing recognition error rates; and (c) the group shuffled co-attention effectively enhances the model’s ability to attend to key speech features, thereby improving overall recognition performance. In summary, while maintaining a reasonable model structure, MSRDA-GSCA improves speech recognition performance and demonstrates strong practical value and promising potential for broader application.

### 4.4. Ablation Experiments on THCHS-30

To investigate the impact of different modules on model performance, two sets of ablation experiments are conducted. The first ablation study focuses on the MSRDA module, and the corresponding results are reported in Table 8. The baseline model achieves a CER of 15.45% and a WER of 20.67%, revealing the inherent performance limitations of the basic architecture. After incorporating the multi-scale fusion convolution (MSD) module, the CER and WER are reduced to 9.59% and 14.22%, respectively, indicating that multi-scale feature extraction plays a substantial role in the effective modeling of speech signals. It is worth noting that the subsequent addition of residual connections (MSRD) yields a CER of 9.24%. This marginal difference between the MSD and MSRD configurations falls within the expected statistical variance of deep acoustic model training, suggesting that isolated residual connections provide limited representational improvements on their own. The progressive ablation results further demonstrate the functional contribution of the components in MSRDA. In particular, the Adaptive Projection Residual Block provides a direct pathway for feature and gradient propagation, facilitating feature reuse and preserving useful acoustic information during successive nonlinear transformations. Removing this pathway forces the acoustic representations to be propagated entirely through the nonlinear transformation layers, which may increase information loss and make network optimization more difficult. This is consistent with the progressive improvement in CER and WER observed across the ablation configurations. Finally, the complete MSRDA model achieves lower CER and WER values of 6.44% and 8.93%, respectively. These results indicate that the discriminability of acoustic features is profoundly enhanced from both acoustic subspace selection and time–frequency localization perspectives only when the multi-scale residual structures are synergistically coupled with the attention mechanism, thereby validating the necessity of this collaborative design.

The ablation study on the GSCA module is presented in Table 9. With the complete GSCA module, the model achieves a CER of 7.32% and a WER of 10.60%, representing reductions of 8.13 and 10.07 percentage points, respectively, compared with the baseline model. This demonstrates that the GSCA module enhances the model’s performance by improving feature attention across multiple dimensions. When the position-wise channel attention is removed, the CER and WER increase slightly to 7.42% and 10.86%, indicating that channel attention plays a critical role in performance improvement, particularly in extracting key signal features. Removing the spatial attention results in a CER of 9.62% and a WER of 14.42%, further confirming that spatial attention is essential for selecting salient time–frequency regions and enhancing recognition accuracy. Finally, when the channel shuffle operation is removed, the CER and WER rise to 11.28% and 15.37%, suggesting that channel shuffle is important for effective information interaction and feature fusion across channels.

In summary, the ablation studies on the MSRDA and GSCA modules further validate the effectiveness and complementarity of each component. The MSRDA module effectively captures multi-scale speech features, enhancing the overall recognition performance of the model. The GSCA module, through its joint channel and spatial attention mechanism, allows the network to more accurately focus on salient feature regions, improving accuracy. The removal of any single module results in performance degradation, demonstrating the critical contribution of both MSRDA and GSCA to the model’s performance. Their combination enables the MSRDA-GSCA model to achieve substantial advantages in speech recognition tasks.

### 4.5. Supplementary Experiments and Ablation Analysis on ST-CMDS

To evaluate the generalization of the proposed framework, comparative experiments were conducted on the ST-CMDS dataset, as summarized in Table 10. The results demonstrate that the proposed MSRDA-GSCA model attains the highest recognition accuracy among all compared architectures, achieving a CER of 10.43% and a WER of 16.47%. This represents a substantial improvement over the baseline DCNN+CTC model, which recorded a CER of 33.42%, and outperforms established models such as DFCNN-Transformer (14.87% CER) and Deep Speech 2 (18.25% CER).

Systematic ablation studies detailed in Table 11 further verify the internal logic of the architecture by examining the individual contributions of its core modules. It is important to note that the ablation progression on the ST-CMDS dataset exhibits a non-monotonic behavior, distinguishing it from the consistent improvements observed on THCHS-30. Specifically, the fundamental MSD configuration achieves a CER of 11.23%, whereas the isolated addition of residual connections (MSRD) and dual-path attention (MSRDA) yields slightly higher CERs of 11.95% and 11.93%, respectively. This indicates that for the shorter, highly variable commands typical of ST-CMDS, locally enhancing complex features through MSRDA alone does not yield immediate benefits and may introduce slight representation bias. However, when the GSCA module is structurally integrated with MSRDA, the performance improves to a CER of 10.43%. This non-monotonic dynamic strongly highlights the synergistic dependency within our collaborative design: the fine-grained, local acoustic features extracted by MSRDA require the global channel shuffling and spatial recalibration provided by GSCA to achieve optimal decoding in complex acoustic scenarios.

Furthermore, a striking dataset-dependent behavior is observed regarding the position-wise channel attention (PCA). In the THCHS-30 evaluation, removing PCA only marginally increases the CER from 7.32% to 7.42%. Conversely, on the ST-CMDS dataset, the “GSCA No PCA” configuration suffers a catastrophic degradation, yielding a CER of 32.18%. We meticulously verified these results and attribute this dramatic discrepancy to the interaction between dataset acoustic variance and the structural placement of PCA.

THCHS-30 consists of clean, laboratory-recorded continuous speech with highly stable feature distributions. In contrast, ST-CMDS comprises mobile recordings from 855 diverse speakers, featuring high acoustic variance and complex environmental redundant acoustic features. Structurally, PCA acts as a critical dynamic filter before the Channel Shuffle (CSM) operation within the GSCA module. Without PCA, uncalibrated channels—which in ST-CMDS often capture severe speaker-specific biases or background artifacts—are directly shuffled and forcibly distributed across all feature groups. This uncalibrated information mixing fundamentally corrupts the semantic consistency of the feature maps, thereby paralyzing the subsequent spatial attention module (SAM). This finding confirms that while pre-shuffle channel recalibration (PCA) is optional for clean and stable speech, it acts as an indispensable safeguard against feature corruption in highly variable, multi-speaker acoustic environments.

### 4.6. Model Complexity Analysis

Table 12 summarizes the parameter counts and inference complexity of several representative speech recognition models, highlighting the architectural efficiency of the proposed framework. The MSRDA-GSCA model utilizes a compact footprint of 4.65 M parameters, which is nearly an order of magnitude smaller than established heavy-weight architectures such as Deep Speech2 (42.94 M) and DFCNN-Transformer (36.55 M). While the proposed model is larger than the minimal DCNN+CTC baseline (1.47 M), it remains more lightweight than hybrid models like LST-RPESR (23.26 M) and TCN-Transformer-CTC (32.08 M). Furthermore, in terms of computational complexity, MSRDA-GSCA requires 72.32 GFLOPs for pure inference, demonstrating higher efficiency than the DFSMN-3-gram (75.20 G) and substantially lower resource consumption than Deep Speech2 (189.35 G). This combination of a low parameter count and optimized FLOPs suggests that the MSRDA-GSCA framework achieves a superior trade-off between model capacity and deployment feasibility on resource-constrained platforms.

**Figure 5:**
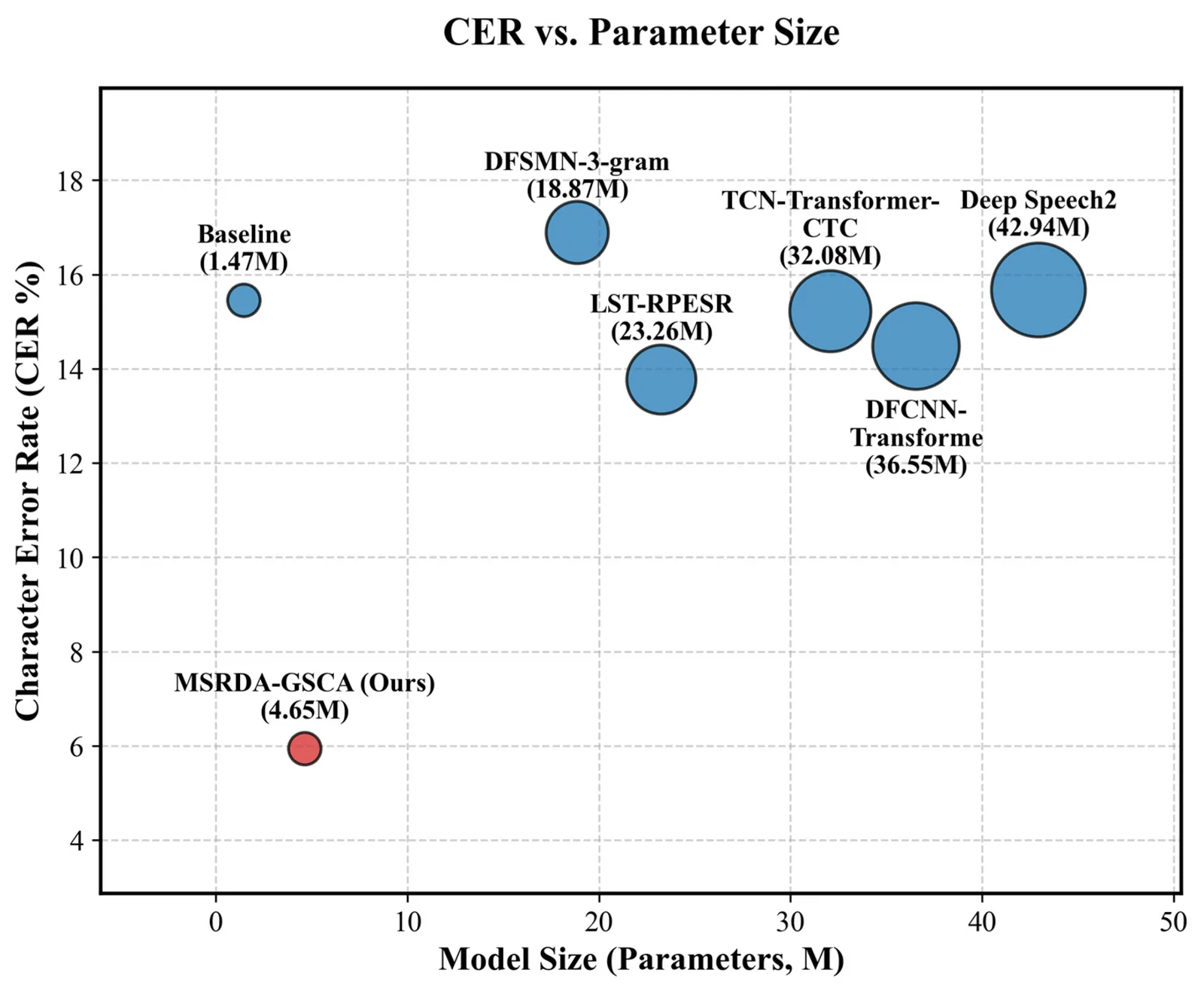
Performance evaluation of MSRDA-GSCA against selected reference models in accuracy and size.

Figure 5 provides a comprehensive visualization of the trade-off between recognition accuracy and model scale for both proposed MSRDA-GSCA and other models. In this bubble chart, the Y-axis represents the CER, the X-axis denotes the number of parameters in millions, and the bubble size correlates with the total number of model parameters. According to the results, our proposed model, MSRDA-GSCA, outperforms all other compared models. With only 4.65 M parameters, it achieves the lowest CER of approximately 6%. In contrast, baseline models and other larger architectures, such as Deep Speech 2 (42.94 M) and DFCNN-Transformer (36.55 M), exhibit much higher error rates despite their larger parameter counts. This comparison demonstrates that our model achieves a superior balance between efficiency and accuracy, providing a high-performance solution with a lightweight architecture.

As shown in Table 13, to further evaluate the practical deployment potential of the proposed framework, we conducted a rigorous Real-Time Factor (RTF) test on an NVIDIA GeForce RTX 3060 GPU. Despite the MSRDA-GSCA model possessing a computational complexity of 72.32 GFLOPs, it achieved a remarkable RTF of 0.0043, with an average inference latency of only 0.0694 s for a standard 16-s audio input. This indicates that the model is approximately 231 times faster than real-time speech, successfully bridging the gap between high-performance feature extraction and efficient inference. These results confirm that our collaborative design effectively optimizes the trade-off between model capacity and execution speed, making it highly suitable for resource-constrained yet time-sensitive ASR tasks. To further evaluate the deployment versatility across non-accelerated platforms, we extended our evaluation to a standard Intel Core i5-12400F CPU. Despite the MSRDA-GSCA model possessing a computational complexity of 72.32 GFLOPs, it maintained a competitive RTF of 0.0332, with an average inference latency of 0.5310 s for a 16-s audio input. This performance indicates that the model remains approximately 30 times faster than real-time speech even on a consumer-grade processor. These results verify that our lightweight architecture and efficient recalibration mechanisms successfully mitigate the computational bottlenecks typically associated with deep acoustic models, ensuring high-performance Mandarin ASR on resource-constrained edge devices without the need for dedicated GPU acceleration.

## 5. Conclusions

In this work, a novel speech recognition model, termed MSRDA-GSCA is proposed, which co-designs a Multi-Scale Residual Deep Convolutional Attention (MSRDA) convolutional network with a Group Shuffled Co-Attention (GSCA) module. By leveraging a parallel multi-scale convolutional architecture and adaptive projective residual connections, the MSRDA module effectively bridges the representation gap between heterogeneous temporal features, ensuring the preservation of fundamental acoustic cues across deep layers. Furthermore, the introduction of a parallel dual-path attention mechanism within MSRDA, optimized by a max-response fusion strategy, recalibrates feature importance with minimal computational redundancy. Complementing this, the GSCA module facilitates global information exchange through a sequential dual-attention pipeline and a channel shuffle operation, effectively mitigating the information isolation typically found in grouped lightweight designs. Experimental evaluations on the THCHS-30 dataset indicate that the MSRDA-GSCA model yields a Character Error Rate (CER) of 5.94% and a Word Error Rate (WER) of 8.32%. Compared to the baseline DCNN+CTC model, these results represent reductions of 9.51 percentage points in CER and 12.35 percentage points in WER. To further verify the generalization capability of the framework, supplementary experiments were conducted on the ST-CMDS dataset, resulting in a CER of 10.43% and a WER of 16.47%. The performance gains observed across both datasets reinforce the effectiveness of the model in handling both formal reading-style speech and informal command-oriented scenarios. Crucially, MSRDA-GSCA achieves these breakthroughs while maintaining high efficiency. Quantitative analysis reveals the model consists of only 4.65 M parameters and requires 72.32 GFLOPs for inference. Most notably, the model achieves an extraordinary Real-Time Factor (RTF) of 0.0043 on a mid-range GPU (NVIDIA RTX 3060) and 0.0332 on a standard CPU (Intel i5-12400F). This favorable trade-off between recognition accuracy and computational cost—achieving over 30× real-time speed even without hardware acceleration—demonstrates the model’s exceptional potential for deployment on resource-constrained edge devices.

In summary, the proposed MSRDA-GSCA model provides an effective and lightweight solution for speech recognition tasks. Future work will focus on extending this framework to larger-scale datasets and exploring more challenging real-world scenarios to further validate its broad applicability.

## Figures and Tables

**Table 1:** Complete implemented architecture.

Stage	Operation	Repeat	Output Size
Input	Log-magnitude spectrogram	1	1600 × 200 × 1
Stage 1	MSRDA; C_out = 32	2	1600 × 200 × 32
Pool 1	Max pool 2 × 2; stride 2	1	800 × 100 × 32
Stage 2	MSRDA; C_out = 64	2	800 × 100 × 64
Pool 2	Max pool 2 × 2; stride 2	1	400 × 50 × 64
Stage 3	MSRDA; C_out = 128	2	400 × 50 × 128
Pool 3	Max pool 2 × 2; stride 2	1	200 × 25 × 128
Stage 4	7 × 7 Conv 128; BN; ReLU	2	200 × 25 × 128
Pool 4	Max pool 1 × 1; stride 1	1	200 × 25 × 128
Stage 5	7 × 7 Conv 128; BN; ReLU	2	200 × 25 × 128
Pool 5	Max pool 1 × 1; stride 1	1	200 × 25 × 128
GSCA	PCA; four-group shuffle; SAM	1	200 × 25 × 128
Reshape	Merge frequency and channels	1	200 × 3200
Classifier	Dense 128; ReLU	1	200 × 128
Output	Dense 1428; Softmax	1	200 × 1428

**Table 2:** Attention configurations and residual ordering.

Component	Configuration
MSRDA branch ordering	Conv; BN; add projection; BN; ReLU
MSRDA channel attention	GAP; C -> max(1, floor(C/16)) -> C
MSRDA spatial attention	1 × 1 Conv C -> 1; sigmoid
Attention fusion	Element-wise maximum of weighted feature maps
GSCA channel attention	128 -> 32 -> 128; reduction ratio 4
GSCA shuffle	4 groups; 32 channels per group
GSCA spatial attention	7 × 7 Conv 128 -> 32 -> 128; BN after each convolution

**Table 3:** Comparison of technical specifications between the THCHS-30 and ST-CMDS datasets.

Characteristics	THCHS-30 Dataset	ST-CMDS Dataset
Releasing Institution	CSLT, Tsinghua University	SurfingTech
Total Duration	>30 h	Approx. 100 h
Linguistic Content	Reading-style (news, classic literature)	Mobile search commands, daily phrases
Corpus Scale	10,000+ utterances	102,600 utterances
Training utterances	10,000	100,000
Development utterances	893	600
Test utterances	2495	2000
Number of Speakers	Multiple (diverse gender, age, and region)	855 speakers
Recording Environment	Quiet laboratory conditions	Relatively quiet mobile interaction
Sampling Rate/Bit Depth	16 kHz/16-bit	16 kHz/16-bit
Auxiliary Resources	LMs, lexicons, and noise-condition test sets	Audio files and corresponding transcriptions
Primary Application	Baseline training and benchmarking	Evaluating command accuracy and generalization

**Table 4:** Mutually exclusive masking branches in the retained implementation.

Probability	Implemented Masking Behavior
60%	No masking.
15%	A sampled temporal interval across all frequency bins.
15%	A sampled frequency interval across all time frames.
10%	Frequency bin 0 within a sampled temporal interval.

**Table 5:** Summary of hardware and software configurations for the experimental environment.

Hardware Configuration	Software Configuration
Intel Core i5-12400F (6C/12T, up to 4.4 GHz)	Linux x86_64 (Ubuntu18.0)
16 GB	CUDA 11.4
NVIDIA GeForce RTX 3060	TensorFlow 2.8.4
12 GB	Python 3.9.19

**Table 6:** Summary of hyperparameter settings for model training.

Parameter	Value
Input shape	1600 × 200 × 1
Output dimension	1428
Optimizer	Adam
Learning Rate	$5\times 10^{-4}$
Batch Size	4
Epsilon	$10\times 10^{-8}$
Epochs	50
Max Label Length	64
Training features	Log-magnitude spectrogram with masking in Section 4.1.3

**Table 7:** Comparison of CER and WER performance across different speech recognition models on THCHS-30.

Model	THCHS-30	
	CER (%)	WER (%)
DCNN+CTC	15.45	20.67
DFSMN-3-gram	16.89	24.62
TCN-Transformer-CTC	15.22	22.26
DFCNN-Transformer	14.48	21.45
Deep Speech2	15.67	22.76
LST-RPESR [35]	13.77	20.66
MSRDA-GSCA	5.94	8.32

**Note:** Results for LST-RPESR are directly extracted from [35]. All other baselines were re-implemented in this study. Notably, DFSMN-3-gram utilizes a 3-gram LM, whereas our proposed method and the baselines utilize a 2-gram LM.

**Table 8:** Ablation Experiment Results of the MSRDA Module on THCHS-30.

Model	THCHS-30	
	CER (%)	WER (%)
DCNN+CTC	15.45	20.67
MSD	9.59	14.22
MSRD	9.24	13.91
MSRDA	6.44	8.93

**Table 9:** Ablation Experiment Results of the GSCA Module on THCHS-30.

Model	THCHS-30	
	CER (%)	WER (%)
DCNN+CTC	15.45	20.67
GSCA No PCA	7.42	10.86
GSCA No SAM	9.62	14.42
GSCA No CSM	11.28	15.37
GSCA	7.32	10.60

**Table 10:** Comparison of CER and WER performance across different speech recognition models on ST-CMDS.

Model	ST-CMDS	
	CER (%)	WER (%)
DCNN+CTC	33.42	38.66
DFSMN-3-gram	30.47	35.70
TCN-Transformer-CTC	20.12	28.64
DFCNN-Transformer	14.87	20.53
Deep Speech2	18.25	24.57
MSRDA-GSCA	10.43	16.47

**Note:** DFSMN-3-gram utilizes a 3-gram LM, whereas our proposed method and the baselines utilize a 2-gram LM.

**Table 11:** Ablation Experiment Results on ST-CMDS.

Model	ST-CMDS	
	CER (%)	WER (%)
DCNN+CTC	33.42	38.66
MSRDA-GSCA	10.43	16.47
MSRDA	11.93	18.24
MSRD	11.95	18.34
MSD	11.23	17.50
GSCA	12.72	19.55
GSCA No PCA	32.18	36.64
GSCA No SAM	14.07	20.98
GSCA No CSM	20.63	27.65

**Table 12:** Comparison of Parameters and Inference Complexity (FLOPs) for Different Speech Recognition Models.

Model	Params	FLOPs
DCNN+CTC	1.47 M	29.97 G
DFSMN-3-gram	18.87 M	75.20 G
TCN-Transformer-CTC	32.08 M	-
DFCNN-Transformer	36.55 M	-
Deep Speech2	42.94 M	189.35 G
LST-RPESR	23.26 M	-
MSRDA-GSCA	4.65 M	72.32 G

**Table 13:** Evaluation of inference latency and Real-Time Factor (RTF) across different hardware platforms.

Inference Device	Avg.Latency (s)	Real-Time Factor (RTF)	Processing Speed
NVIDIA RTX 3060	0.0694	0.0043	231× Faster than Real-time
Intel i5-12400F	0.5310	0.0332	30× Faster than Real-time

## Data Availability

THCHS-30: https://www.openslr.org/18/ (accessed on 8 July 2024). ST-CMDS: https://www.openslr.org/38/ (accessed on 24 September 2024). The source code for MSRDA-GSCA is publicly available at https://github.com/leilvling/MSRDA-GSCA.

## Abbreviations

The following abbreviations are used in this manuscript:

MSRDA	Multi-Scale Residual Deep Convolutional Attention
MSRD	Multi-Scale Residual Deep Convolution
MSD	Multi-Scale Deep Convolution
GSCA	Group Shuffled Co-Attention
CER	Character Error Rate
WER	Word Error Rate
ASR	Automatic Speech Recognition
DNNs	Deep Neural Networks
CNNs	Convolutional Neural Networks
RNNs	Recurrent Neural Networks
CTC	Connectionist Temporal Classification
MCNNs	Multi-Scale Convolutional Neural Networks
ResNets	Residual Networks
RAGLU	Residual Attention Gated Linear Unit
DCNNs	Deep Convolutional Neural Networks
BN	Batch Normalization
PCA	Position-wise Channel Attention Modeling
CSM	Channel Shuffling and Global Information Mixing
SAM	Spatial Attention Modeling
